# Preoperative primary tumor SUVmax on PET/CT correlates with Breslow thickness and is associated with survival in melanoma: A retrospective cohort study

**DOI:** 10.1016/j.jdin.2025.10.003

**Published:** 2025-10-18

**Authors:** Yi-Han Chang, Yi-Hsuan Huang, Shin-Chen Pan, Julia Yu-Yun Lee, Cheng-Lin Wu, Tak-Wah Wong, Hsi-Huei Lu, Wei-Ting Liu

**Affiliations:** a Department of Dermatology, National Cheng Kung University Hospital, College of Medicine, National Cheng Kung University, Tainan, Taiwan; b Skin Cancer Team, National Cheng Kung University Hospital, College of Medicine, National Cheng Kung University, Tainan, Taiwan; c Department of Oncology, National Cheng Kung University Hospital, College of Medicine, National Cheng Kung University, Tainan, Taiwan; d Department of Surgery, Section of Plastic and Reconstructive Surgery, National Cheng Kung University Hospital, College of Medicine, National Cheng Kung University, Tainan, Taiwan; e Department of Pathology, National Cheng Kung University Hospital, College of Medicine, National Cheng Kung University, Tainan, Taiwan; f Department of Biochemistry and Molecular Biology, College of Medicine, National Cheng Kung University, Tainan, Taiwan; g Center of Applied Nanomedicine, National Cheng Kung University, Tainan, Taiwan; h Division of Nuclear Medicine, Department of Medical Imaging, National Cheng Kung University Hospital, College of Medicine, National Cheng Kung University, Tainan, Taiwan

*To the Editor:* Melanoma is an aggressive skin cancer and a major cause of cancer-related mortality.^1^ Acral lentiginous melanoma (ALM), while uncommon globally, is the most prevalent subtype in Asian populations and often presents with advanced disease due to delayed detection.^2^

18F-fluorodeoxyglucose (18F-FDG) positron emission tomography/computed tomography (PET/CT) is widely used for staging advanced melanoma,^3^ but not routinely recommended for early-stage disease. In Taiwan, PET/CT is reimbursed for all melanoma patients regardless of stage, providing a unique opportunity to explore its preoperative role, especially in ALM where large lesions often preclude complete excisional biopsy and preoperative staging may be uncertain.

We retrospectively reviewed 95 patients with histologically confirmed melanoma at a tertiary center in Taiwan (2015-2022). Among them, 64 (67.4%) underwent PET/CT; 31 (32.6%) having preoperative scans with >50% residual primary tumors were included for final analysis (Table I). The median age was 75 years (range:44-92); 64.5% were male and 67.7% had ALM. ALM and non-ALM demographics were comparable (Supplementary Table I, available via Mendeley at http://doi.org/10.17632/5wwx5w8rh4.1). All patients underwent incisional biopsy followed by PET/CT after a minimum 2-week interval (mean, 24.8 days; range, 14-77 days). Biopsy-excision depth discrepancies occurred in 54.8% of cases.

**Table I:** Demographics and tumor characteristics of analyzed cohort

Parameters	Preoperative PET/CT∗ (*n* = 31)	Entire cohort (*n* = 95)
Age, y, median (range)	75 (44-92)	66 (26-94)
Men, *n* (%)	20 (64.5%)	50 (52.6%)
Acral melanoma, *n* (%)	21 (67.7%)	56 (58.9%)
T stage, *n* (%)		
Tis	0	22 (23.2%)
T1	1 (3.2%)	9 (9.5%)
T2	7 (22.6%)	11 (11.6%)
T3	8 (25.8%)	17 (17.9%)
T4	15 (48.4%)	25 (26.3%)
Tx	0	11 (11.6%)
Final stage		
Stage 0	0	23 (24.2%)
Stage 1	6 (19.4%)	16 (16.8%)
Stage 2	14 (14.7%)	25 (26.3%)
Stage 3	10 (32.3%)	22 (23.2%)
Stage 4	1 (3.2%)	9 (9.5%)
Breslow thickness, mm, median (range)	4 (1-12)	3 (0.3-21)
Type of skin biopsy, *n* (%)		
Incisional	31 (100%)	69 (72.6%)
Excisional	0	17 (17.9%)
Unknown or not performed	0	9 (9.5%)
Biopsy-excision depth discrepancy, *n* (%)	17 (54.8%)	22 (23.2%)
SLNB/CLND performance, *n* (%)		
SLNB	16 (51.6%)	35 (36.8%)
CLND	7 (22.6%)	13 (13.7%)
Not done	8 (25.8%)	47 (49.5%)
SUVmax of primary tumor, mean (SD)	4.18 (3.24)	NA

*CLND,* Complete lymph node dissection; *SLNB,* sentinel lymph node biopsy.

∗ Biograph mCT Flow PET/CT system (Siemens), featuring an axial spatial resolution of 4.4 mm measured at 1 cm vertically.

We investigated the correlation between primary tumor SUVmax and final Breslow thickness. Linear regression showed a moderately positive correlation across all melanoma types (r = 0.658, *P* < .001), and a stronger correlation in ALM (r = 0.828, *P* < .001) (Fig 1). Subgroup analysis showed higher correlation in advanced-stage melanoma (r = 0.618) compared with early-stage disease (r = 0.585) (Supplementary Fig 1, available via Mendeley at http://doi.org/10.17632/5wwx5w8rh4.1). Receiver operating characteristic analysis identified an SUVmax threshold of 4.945 predictive of overall survival. Kaplan–Meier analysis revealed significantly worse survival in patients with SUVmax ≥4.945 (*P* = .04, Generalized Wilcoxon method) (Supplementary Fig 2, available via Mendeley at http://doi.org/10.17632/5wwx5w8rh4.1).

**Fig 1:**
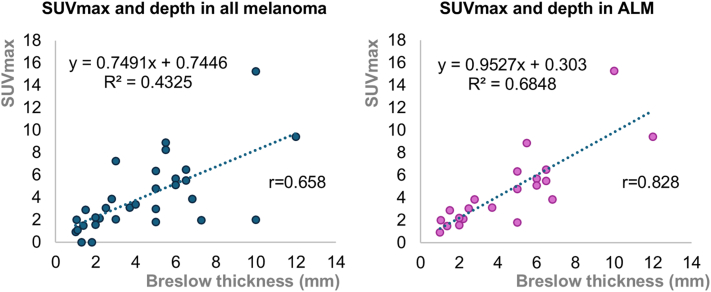
Linear regression analysis showing the correlation between the maximum standardized uptake value (SUVmax) and Breslow thickness across all melanoma cases (r = 0.658, *P* < .001), and an even stronger correlation in acral lentiginous melanoma (ALM) cases (r = 0.828, *P* < .001).

Previous studies highlighted frequent biopsy-excision depth discrepancies in melanoma,^4^ a finding also observed in our study. This is particularly relevant in ALM, where excisional biopsy is often impractical, potentially leading to understaging and inadequate surgical planning. PET/CT could provide complementary information for preoperative decision-making. Our findings suggest that high primary tumor SUVmax may warrant consideration of wider margins or sentinel lymph node biopsy, when biopsy adequacy is uncertain. Although alternative imaging modalities such as high-frequency ultrasonography have shown potential in predicting Breslow thickness,^5^ ALM cases are underrepresented or excluded in these studies.

While the prognostic value of PET parameters is well studied in advanced melanoma, few investigations focus on primary tumor, particularly in ALM-predominant regions. Our results suggest that preoperative SUVmax offers prognostic value. However, the area under the curve was only 0.577, which is marginally better than chance. These exploratory results should therefore be interpreted with caution.

Limitations include the retrospective, single-center design, small sample size, and inclusion of only patients with >50% residual primary melanoma, which may have introduced bias. Nonetheless, this study provides novel insights into the clinical utility of primary tumor SUVmax in an ALM-predominant cohort.

In conclusion, preoperative PET/CT SUVmax of primary melanoma correlates with Breslow thickness and is associated with survival. In ALM, PET/CT may serve roles beyond metastasis screening, aiding surgical decision-making. Prospective validation is warranted.

## Conflicts of interest

None disclosed.
